# Three-dimensional non-destructive soft-tissue visualization with X-ray staining micro-tomography

**DOI:** 10.1038/srep14088

**Published:** 2015-09-25

**Authors:** Juliana Martins de S. e Silva, Irene Zanette, Peter B. Noël, Mateus B. Cardoso, Melanie A. Kimm, Franz Pfeiffer

**Affiliations:** 1 Lehrstuhl für Biomedizinische Physik, Physik-Department & Institut für Medizintechnik, Technische Universität München, 85748 Garching, Germany; 2 Brazilian Synchrotron Light Laboratory, 13083-970, Campinas, Brazil; 3 Diamond Light Source, Harwell Science and Innovation Campus, Didcot, OX110DE, United Kingdom; 4 Department of Radiology, Technische Universität München, 81675 München, Germany

## Abstract

Low inherent contrast in soft tissues has been limiting the use of X-ray absorption micro-computed tomography (micro-CT) to access high-resolution structural information of animal organs. The staining agents used in micro-CT to improve the contrast fail in providing high-quality images of whole organs of animals due to diffusion problems of the staining agent into the sample. We demonstrate a staining protocol that incorporates a biochemical conditioning step prior to exposure to the staining agent that succeeds in overcoming the diffusion problems, thus quickly providing high-quality micro-CT images of whole organs of mammals. Besides of yielding non-distorted three-dimensional information at the same spatial resolution accessible in histological sections, micro-CT images of whole organs stained by our method enable easy screening of slices along any direction of the volume thus demonstrating new possibilities of structural analysis in biomedical science.

Detailed morphological visualization of soft tissues at the micrometer scale is essential for functional, comparative and developmental studies^1^,^2^,^3^,^4^. Moreover, accurate histopathological analysis of potentially affected tissue is crucial for the investigation and treatments of diseases^5^,^6^. However, none of the imaging methods existing nowadays can easily provide high-contrast volumetric images of the investigated specimen with isotropic micrometer spatial resolution. Staining combined with histological sectioning yields high-resolution (sub-micrometer) information in the sectioning plane. The possibility of building an entire volume from serial histological slices is widely investigated^7^,^8^, but still remains very challenging and is usually very time-consuming. Moreover, procedures of both data acquisition and analysis require intensive manual interaction, while sample distortions arising in the slicing process - such as holes, foldings and missing parts - can hardly be compensated^9^. Micro-magnetic resonance imaging (microMRI) provides high contrast data with isotropic spatial resolution^2^. However, microMRI setups are generally very expensive and the resolution is currently limited to tens of micrometers by the image forming method. X-ray absorption micro-computed tomography (micro-CT) can also be used and provides three-dimensional (3D) information, showing increased resolution down to sub-micrometer scale. In the biomedical field, micro-CT is used to investigate strongly absorbing samples, such as bones or teeth, but fails in visualizing features which have similar and weak X-ray absorption properties, such as different types of soft tissues. Recently, nanometric contrast agents has been used to increase the contrast both in CT and MRI^10^,^11^. Their architecture can be optimized to enable their use as multimodal imaging agents, thus showing clear advantages over the contrast agents regularly used in CT and MRI. However, their complex synthetic procedures strongly increases the production costs and limits their use.

To overcome the limitations of X-ray absorption micro-CT, several X-ray phase-contrast imaging techniques have been developed in the last decades^12^,^13^. However, these methods often require a sophisticated setup and very special X-ray beam characteristics, which in most of the cases can be found only at large scale synchrotron facilities. Thus, while phase-contrast X-ray imaging methods perform extremely well in 3D visualization of low absorbing features at the micrometer and sub-micrometers scale, their implementation, access, and widespread use are severely limited by the complexity of the setup.

Staining the sample with appropriate chemical agents is a simple way of increasing the contrast of relevant low-absorbing features in the specimen. This procedure is the core of histological investigations and is also often used in microMRI. However, only very recently staining for micro-CT has been demonstrated to be a valid tool for increasing the contrast in non-mineralized biological tissues. In the earliest works, this technique has been applied to visualize, among others, development in chick embryos^14^. More recently, the staining technique has been used to increase soft-tissue contrast in micro-CT images of samples of larva^15^, young mice forelimbs^16^, mammals hearts^17^ and heart parts^18^.

One issue repeatedly described in several works in this area is related to the slow diffusion of the staining agent into the samples. The protocols that succeed in providing high-quality micro-CT images are those which are able to overcome this diffusion issue. They are characterized by long staining times, of one^18^ or more weeks^19^,^20^, or by the use of highly concentrated staining solutions^17^,^21^, which can cause intense sample shrinkage^22^. Some agents are described to be adequate only for samples with limited epidermal layers^23^. Similarly, good results are also achieved when staining is performed over young tissues^24^ or over samples that had the epidermal layers removed^21^. Here, we now report on a novel staining protocol that successfully overcomes the current limitations of present staining approaches and solves particularly the issue of slow diffusion through the epidermis of animal organs. The main breakthrough of our approach relies on a combined biochemical conditioning and staining procedure for the tissue sample, which enables the diffusion of the stained molecules into the specific tissue. In the following, we demonstrate how our new protocol can efficiently yield volumetric and high-contrast visualization of 3D tissue samples. We validate our method by applying it to several animal organs, and 3D data with spatial resolution comparable to those obtained in histology sections are presented.

## Results

**Figure 1:**
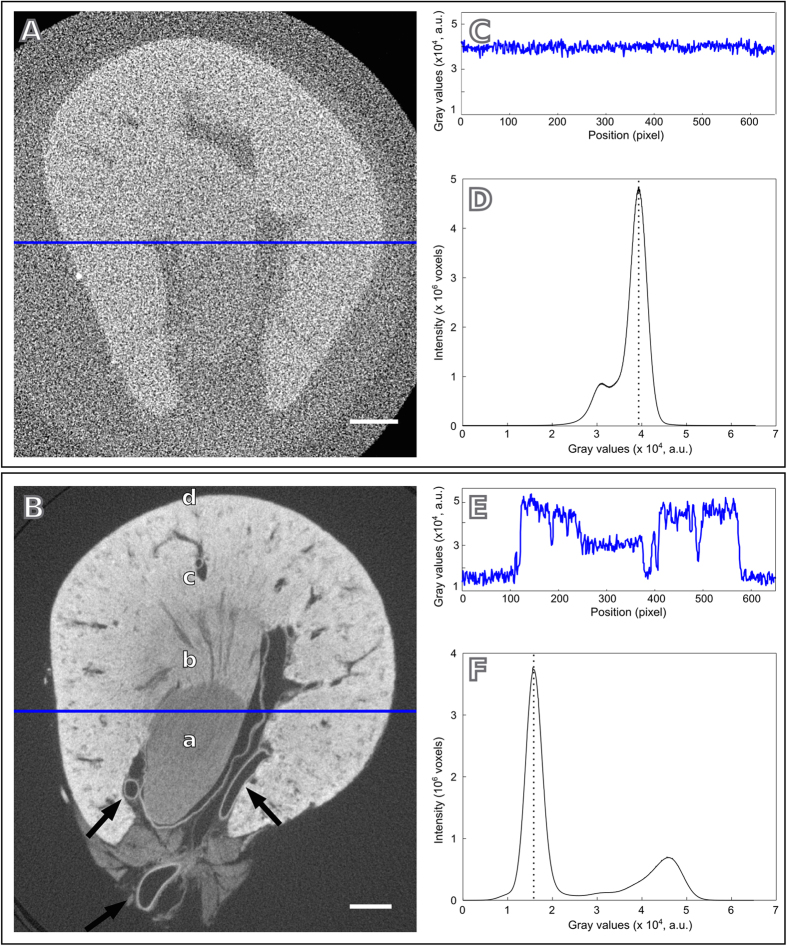
Comparison between non-stained and iodine-stained X-ray micro-CT images of a mouse kidney using the graded ethanol concentration fixation (GECF) protocol. The staining procedure increases the absorption contrast, as confirmed by comparing the tomographic slices of (**A**), a non-stained kidney, and (**B**), an GEFC iodine-stained kidney. Kidney structures are well delineated in (**B**) and a) the inner medulla, b) the inner stripe and c) the outer stripe of the outer medulla, d) the renal cortex and some veins and arteries (arrows) are clearly identified. Profiles along the blue path in (**A**,**B**) are shown in (**C**,**E**), respectively. Distribution of voxel intensity values are shown in (**D**) for the non-stained kidney and in (**F**) for the GEFC-stained kidney. Scale bars: 500 *μ*m. The images show one out of 1000 slices obtained from these scans.

To show the broad applicability of our method, a large variety of mouse organs (kidney, liver, testicle, heart, lungs, brain, stomach, pancreas and spleen) were studied with our newly developed protocol, which essentially comprises a fixation procedure in a graded dehydration series of ethanol (short: graded ethanol concentration fixation - GECF) followed by a subsequent staining with iodine (see methods section for more details). Figure 1 compares tomographic slices obtained under the same experimental conditions of a non-stained mouse kidney (Fig. 1A) and a kidney stained with iodine using our new protocol (Fig. 1B). The visual comparison of these data highlights the tremendous increase in contrast achieved by the staining procedure. While in the non-stained image the sample appears only with a faint contrast, our GECF staining method yields visualisation of a considerably higher amount of details in the micro-CT slice (Fig. 1B).

The increase in contrast produced by our staining protocol can be verified by comparing the gray value profiles in Fig. 1C,E. Significantly distinct intensity variations of the gray values are observed between stained and non-stained samples. Distributions of the voxel intensity values are shown in the histograms of Fig. 1D,F, where the peaks related to the background are indicated by the dotted lines. The histograms are consistent with the wider range of intensity values observed for the stained kidney with respect to the non-stained counterpart. The voxel gray value distribution of the non-stained kidney is superimposed by the background signal (Fig. 1D), while in the histogram of the stained kidney (Fig. 1F) the gray values distribution of the sample is distinct from the background signal and is seen in the right side of the plot.

**Figure 2:**
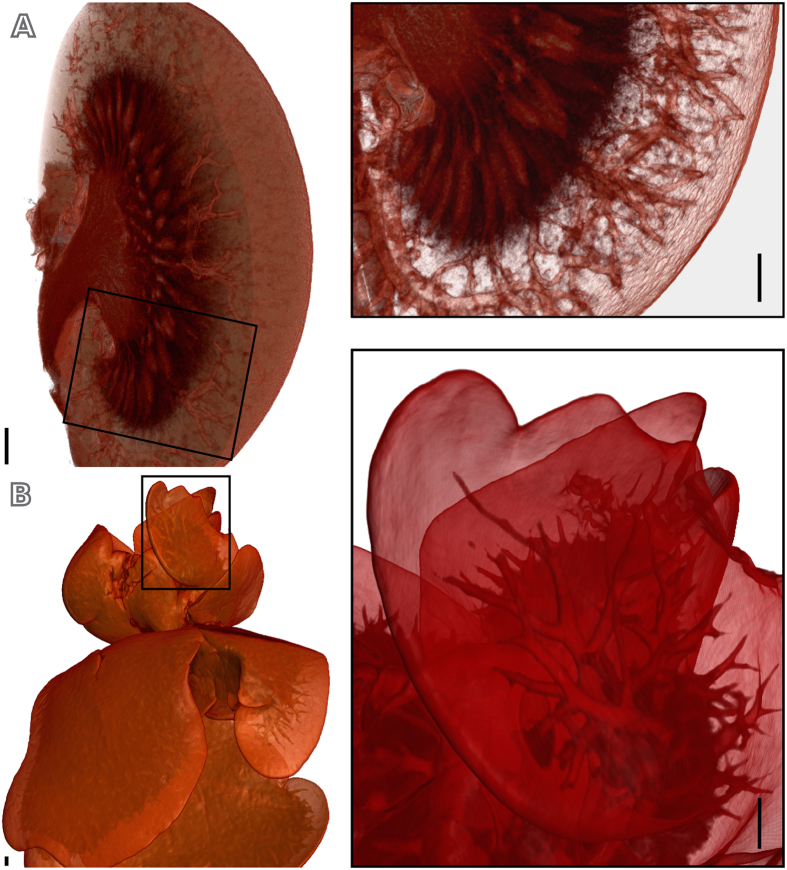
Three-dimensional renderings of GEFC-stained micro-CT scans of different mouse organs. GECF-stained mouse organs, where black rectangles delineate the regions enlarged to show details. (**A**) Virtual cut through the volume of a kidney, showing inner and outer medullas, as well as the renal vascular structure; (**B**) liver lobes, where the profile of the veins of approximately 150 *μ*m diameter or less is seen. Scale bars: 500 *μ*m.

Due to the excellent contrast and the high resolution produced by micro-CT imaging of the stained sample, an intensity-based segmentation of the reconstructed volume separating the different tissues in the organ was possible. A virtual section through the volume of the stained kidney (Fig. 2A) reveals the organ structures and the texture of its surface. The highlighted and enlarged area illustrates the significantly rich structural information available. The inner medulla is pseudo-colored in a darker tone and the outer medulla was set transparent to allow the visualization of the renal vascular structure, with vessels in the micrometer scale colored in red in the figure.

The potential of our protocol in imaging organs of animals is also illustrated in the 3D rendering of a mouse liver (Fig. 2B). By modulating the image transparency, besides of seeing the liver lobes profile, details in the vascular structure of the mouse liver were revealed. Similar analysis was performed for a variety of other mouse organs, such as brain, heart, lungs, between others (see supplementary material).

**Figure 3:**
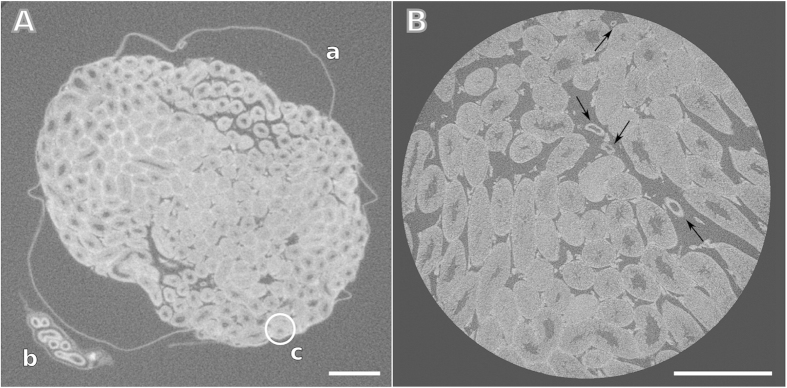
Tomographic slices obtained from imaging a GEFC-stained mouse testicle with two different micro-CT scanner setup geometries. The features visible in the image with moderate resolution (pixel size: 7.6 *μ*m) in (**A**) include a) tunica albuginea; b) epididymis and c) seminiferous tubules (the circle highlight one tubule). In the higher resolution image shown in (**B**) (pixel size: 1.9 *μ*m), the organization of the seminiferous tubules is revealed with higher level of detail, as well as some vessels indicated by the arrows. Scale bars: 500 *μ*m.

Benchtop micro-CT scanners are versatile and volumes at different length scales of the same specimen can be achieved by simply changing the geometry of the setup to enable 3D moderate- and high-resolution visualization of the sample. Tomographic slices of a mouse testicle in moderate and high-resolution are shown in Fig. 3. A series of structures, including the seminiferous tubules, epididymis and tunica albuginea is already well visualized in the moderate resolution tomographic slice of the testicle (Fig. 3A). The high-resolution tomographic slice (Fig. 3B) reveals a more accurate view of the organization and the morphology of the seminiferous tubules with a diameter of approximately 150 *μ*m, as well as some blood vessels indicated by arrows.

**Figure 4:**
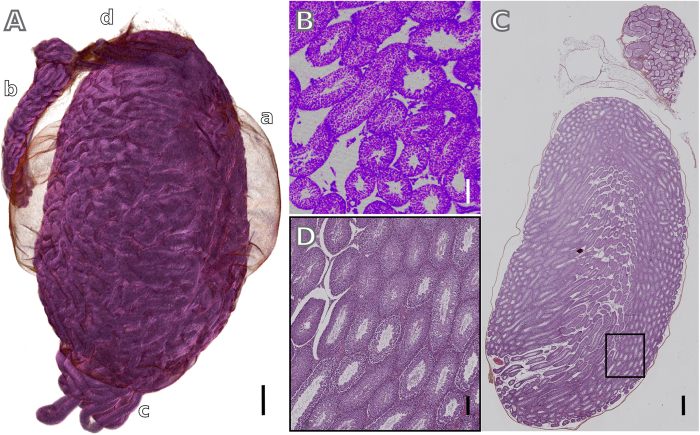
Comparison between the stained micro-CT and histological images. (**A**) Three-dimensional rendering of the whole mouse testicle, showing a) tunica albuginea; b) epididymis, c) seminiferous tubules and d) adipose tissue. A virtual axial cut of a high-resolution volume is shown in (**B**) and the features observed in this virtual histology section correlate well with those seen in the hematoxylin and eosin stained section in (**C**,**D**). Scale bars: 500 *μ*m for (**A**,**C**); 100 *μ*m for (**B**,**D**).

The three-dimensional rendering shown in Fig. 4A was produced from the moderate-resolution micro-CT dataset and has quality similar to volumes obtained from phase-contrast X-ray data^25^. Furthermore, the structures exhibited in the volume are the same seen in the hematoxylin and eosin stained section of the mouse testicle (Fig. 4C). In fact, the testicle structures visible in the enlarged histological image (Fig. 4D) correlates well with the image produced by micro-CT (Fig. 4B). A closer look to both images reveals structures organized in the tubules walls, which appear to be spermatogonia cells and primary spermatocytes.

## Discussion

In this study, a number of animal organs with variable dimensions obtained from a mouse was visualized by applying a newly developed GECF staining protocol in combination with high-resolution micro-CT scanning. The excellent image contrast produced by applying this method was demonstrated in the quantitative analysis of micro-CT slices of a sample before and after staining (Fig. 1). This great contrast improvement enabled us to obtain volumes of all organs imaged (Figs 2 and 4 and supplementary material) revealing, besides of the organs morphology, the precise organization of their structures in 3D.

In micro-CT, by simply changing the geometry of the experimental setup, volumes with different resolutions can be obtained. Using a moderate-resolution setup we were already able to visualise the whole organisation of vessels of less than 150 *μ*m in the mouse kidney, liver and lungs; the texture of the esophagus epithelium; and the organisation of seminiferous tubules in mouse testicle, just to list some structures among others. Images with increased level of details were obtained throughout the high-resolution setup. For example, the image of the mouse testicle obtained using this type of setup gives relevant morphologic information, which is comparable to those obtained by highly evolved specialised methods, such as X-ray grating-based phase tomography at high-brilliance synchrotron sources^25^. Furthermore, the resulting images have a resolution similar to conventional histology (Fig. 4), with the additional benefit of providing non-distorted and fast 3D information. The staining method presented here yielded micro-CT images of a mouse kidney and a mouse cerebellum at a level of detail that is only available from other multiple sectioning techniques or highly sophisticated large-scale-facility-based X-ray methods^26^,^27^. Probably the biggest advantage over other lab-based techniques is the relatively fast (few hours) data acquisition, and the non-destructive and non-distorted 3D imaging visualisation. As X-ray phase-contrast imaging techniques, our method is also able to provide volumetric images, however it does not require sophisticated setups, special X-ray beam characteristics or a trained user, being therefore much more accessible to the general scientific community.

Compared to previous attempts with X-ray staining agents, which are very mostly applied to animal samples without skin or samples with limited epidermis (like embryos and young animals), with our approach we were able to successfully obtain high-quality volumes of several mammals organs without using any of these strategies. Our staining protocol challenges previous procedures in that it incorporates a crucial biochemical conditioning step prior to the actual exposure to the staining agent. The presented GECF method uses ethanol in a specific dehydration step that increases cells membrane permeation^28^ and, therefore, the stain molecules can diffuse more easily into the animal tissues.

Adult animal organs can be difficult to stain because the permeability of animal tissue decreases as the epidermis develops^21^,^29^. Large samples, with numerous layers, also suffer from low permeation of the stain molecule into their core, which can originate heterogeneities in the diffusion. With our protocol, we were able to quickly stain whole organs of animals. Starting from a sample already fixed in formaldehyde, the whole protocol takes less than 24 h, approximately the same time described elsewhere to stain samples lacking epidermis^14^,^30^. After micro-CT analysis of the samples, which takes approximately 2 h, a 3D volume generated images with the same resolution accessible in histological sections.

In conclusion, the results obtained through our GECF staining protocol provide rich morphological information and structures at the micrometer length scale can be easily discriminated. Besides of yielding 3D information at the micrometer scale without inserting artifacts produced by a physical sectioning of the sample, the clear advantage of the staining method presented here over histology is that the specimen preparation protocol is less tedious, much less time-consuming and it does not require cutting the sample. Moreover, the information provided by micro-CT of samples stained by our method not only enables quantitative 3D image analysis but also allows quick and easy screening of slices along any direction of the volume. As the spatial resolution of the method is currently virtually limited only by the capabilities of the micro-CT machine, subcellular resolution may be achieved with this method. While here our method has been demonstrated on specimens which do not show any pathological sign, the same procedure could equally be applied to investigate diseased specimens to, e.g., locate with high precision the diseased region as well as to study volumetric extension and properties of the pathological areas.

## Methods

### Sample preparation

Mouse organs (kidney, testicles, brain, liver, lung, heart, stomach and spleen) derived from a male, 4 week old C57Bl/6 mouse (Taconic, Denmark). Before organs removal, animal housing was carried out as determined in the European Union guidelines 2010/63 at the Centre for Preclinical Studies (ZPF) at the Klinikum rechts der Isar - TU München. The mouse was sacrificed by cervical dislocation under isoflurane anaesthesia, consequently avoiding suffering, according to § 4, Abs. 3 of the German Animal Protection Law. Samples were fixed in 4% formaldehyde in phosphate buffer solution (PBS 1X). After 2 days of fixation, the mouse organs were dehydrated in ethanol. To prevent a further and abrupt shrinkage of the fixed sample, a graded series of ethanolic solutions was used, beginning with 50% ethanol for 1 h and the following ethanol solutions in the concentrations listed, for 1 h each: 70, 80, 90, 96 and 100%. After, the mouse organs were stained in 1% wt. I_2_ solution in absolute ethanol^14^ for 14 h. Samples were washed in absolute ethanol and then placed in 2 mL plastic container filled with absolute ethanol for scanning.

### Data acquisition

Samples were scanned on a Zeiss Xradia Versa 500 system (www.xradia.com) with the X-ray source operating with anode voltage at 50 or 70 kV and power between 3 and 5 W. Different combinations of source-sample and sample-detector distances were used to fit the entire region of interest in the field-of-view (see supplementary material). X-ray detection of the projection image was performed with scintillator crystals (Xradia Inc.). The light was focused by a Nikon microscope objective lens and detected via a CCD cooled camera. Images were acquired with exposure times between 1 to 10 s and 1601 to 2001 projections over 360° and detector binning of 2. Pixel sizes at the sample plane varied from 2 to 27 *μ*m. Images were reconstructed with the volume reconstruction software integrated in the Xradia machine and the 3D renderings presented here were created using the commercial software VGstudio (www.volumegraphics.com). Volumetric renderings were done using Phong or Scatter HQ algorithms.

## Additional Information

**How to cite this article**: Silva, J. M. S. *et al.* Three-dimensional non-destructive soft-tissue visualization with X-ray staining micro-tomography. *Sci. Rep.* **5**, 14088; doi: 10.1038/srep14088 (2015).

## Supplementary Material

Supplementary Information
