# Supplementary material for: Three-dimensional non-destructive soft-tissue visualization with X-ray staining micro-tomography
Source: Sci Rep. 2015 Sep 25;5:14088. doi: 10.1038/srep14088 (PMC4585898; doi:10.1038/srep14088)
Supplement: Supplementary Information [file srep14088-s1.pdf]

Three-dimensional non-destructive soft-tissue visualization with X-ray staining micro-tomography - Supplementary Information

Juliana Martins de Souza e Silva[1,2*], Irene Zanette[1,3], Peter B. Noël[1,4], Mateus B. Cardoso[2], Melanie A. Kimm[4], Franz Pfeiffer[1,4*]

[1] Lehrstuhl für Biomedizinische Physik, Physik-Department & Institut für Medizintechnik, Technische Universität München, 85748 Garching, Germany
[2] Brazilian Synchrotron Light Laboratory, 13083-970, Campinas, Brazil
[3] Diamond Light Source, Harwell Science and Innovation Campus, Didcot, OX11 0DE, United Kingdom
[4] Department of Radiology, Technische Universität München, 81675 München, Germany
*e-mail: juliana.martins@tum.de & franz.pfeiffer@tum.de

Three-dimensional rendering of several stained organs.

We tested the graded ethanol concentration fixation (GECF) staining protocol in a number of organs removed from mice. Immediately after removal, organs were fixed in formaldehyde in phosphate buffer solution and further treated with ethanol. Ethanol is known to increase cells membrane permeation[1]. Thus, after treating the samples with ethanol, cells membranes offer no more extreme opposition to the iodine diffusion. Dehydration with ethanol is expected to favor the staining of adult animals organs, which are composed of cells much less permeable than those of embryos organs. Also, a large diffusion barrier is present in samples because of numerous layers to penetrate through until reaching the organ core.

All stained samples presented in this work were treated with iodine. Iodine solution stains the animal organs as a result of the formation of a complex of this stain and the glycogen present in animal tissues[2]. Therefore, by interacting with glycogen, which is the carbohydrate reserve in most of animal cells[2], iodine is able to bind to the animal soft-tissue and to stain the whole sample. Here we presented results of micro-CT imaging of small mouse organs stained with iodine. In fact, the staining molecule diffused homogeneously in all samples and a clear differentiation between a number of structures in the mature animal organs was therefore possible.

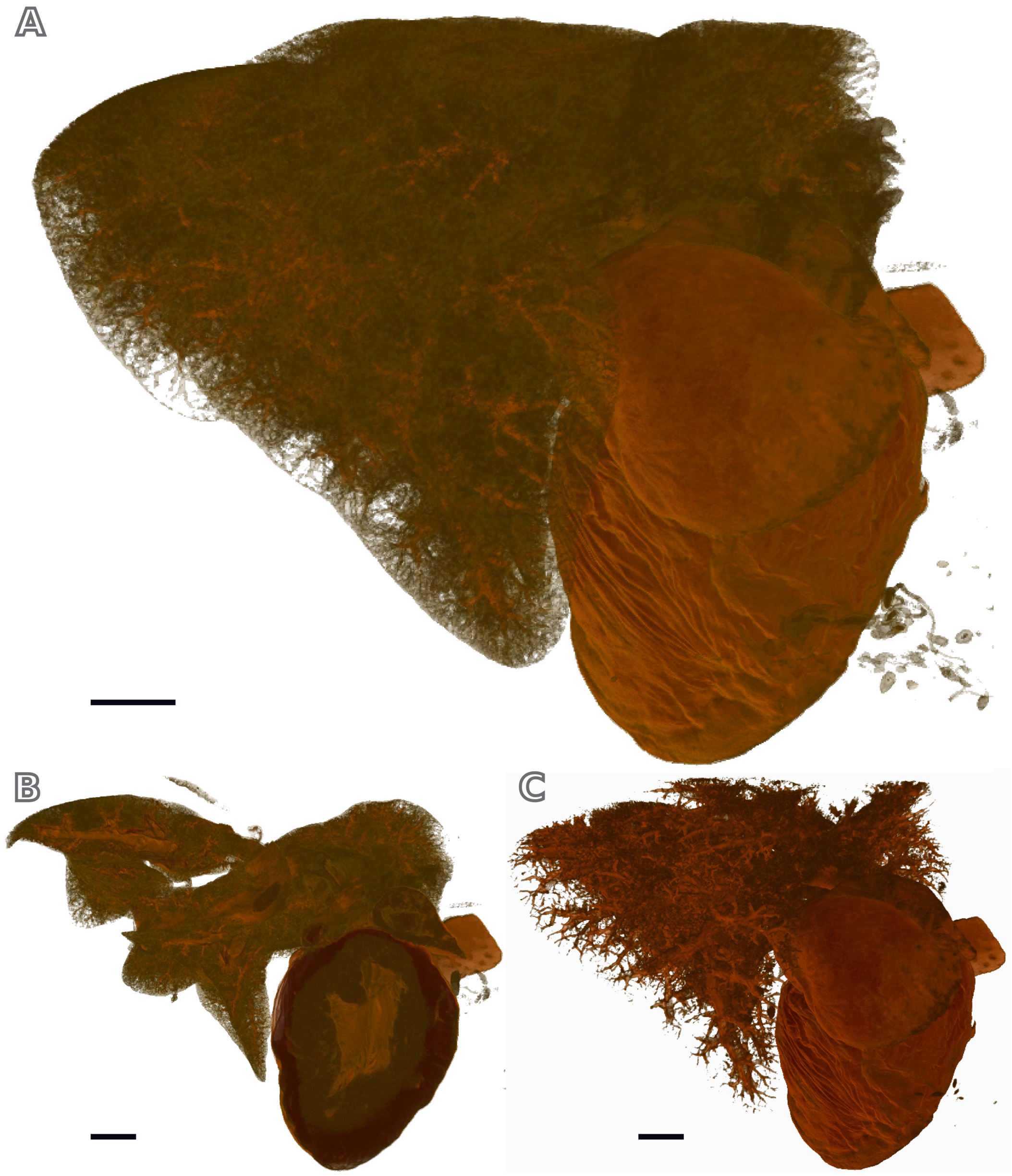

Supplementary Figure 1: **Volumetric rendering of heart and lungs of a mouse.** Mouse heart and lungs are shown in (A). The sample was virtually cut to show heart cavity in (B). By modulating the images transparency and color, different structures can be highlighted. For example, in (C), the whole pulmonary vascular structure is highlighted. Scale bars: 1000 μm.

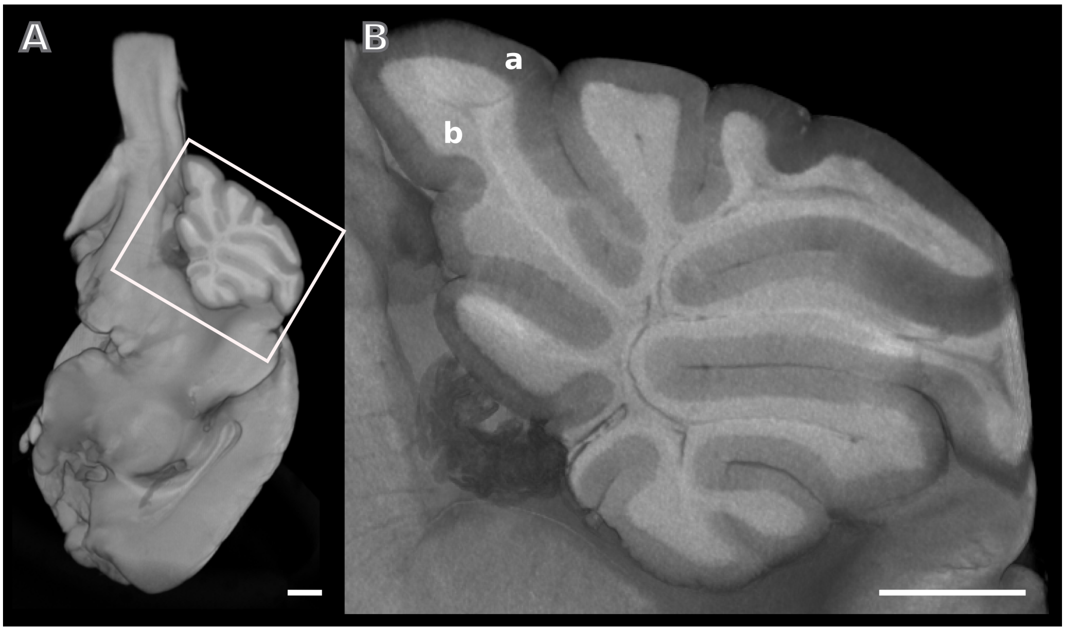

Supplementary Figure 2: **Volumetric rendering of a mouse brain.** (A) Midsagittal cut of a whole mouse brain. The area in the rectangle in (A) corresponds to the cerebellum shown in (B). The enlarged image of the cerebellum shows clear separation between a) gray and b) white matter areas. Images are similar to those obtained by X-ray grating-based phase tomography [3]. Scale bars: 1000 μm.

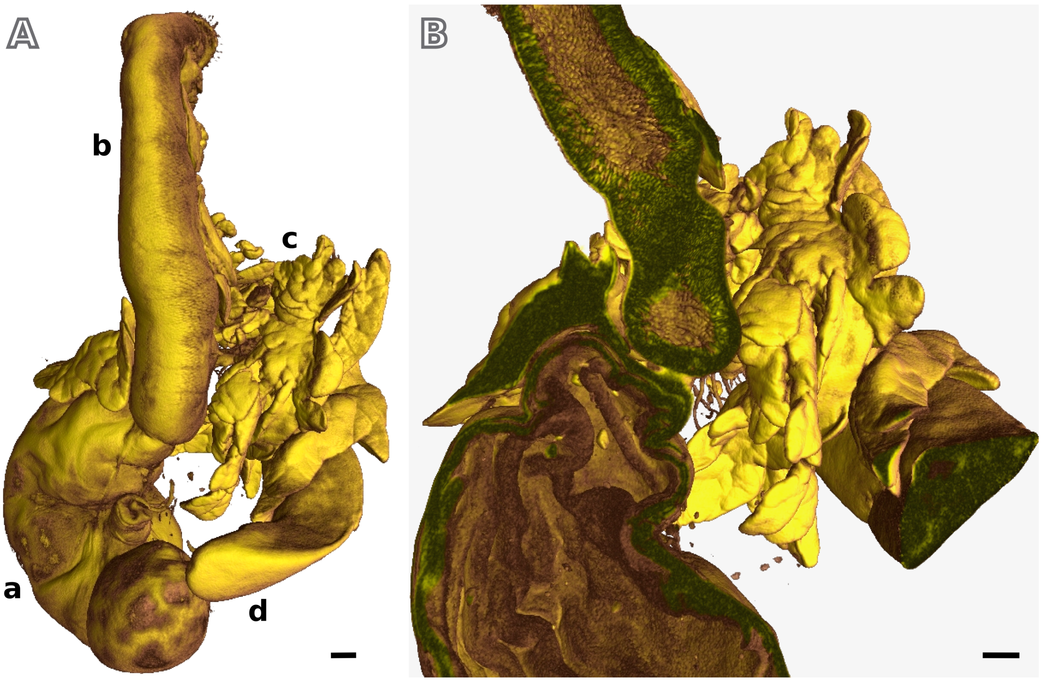

Supplementary Figure 3: **3D geometry of a mouse digestive system, spleen and pancreas.** Whole organs are shown in (A): a) stomach, b) esophagus, c) pancreas and d) spleen. The virtual cut in (B) exhibit the stomach cavity and the highly structured esophagus wall, with its squamous epithelium. Scale bars: 1000 μm.

Table 1: **Micro CT experimental conditions.** Several mouse organs were imaged using different setups to fit the appropriate region of interest and for good image statistics.

Sample	Voltage/Power (kV/W)	Optical magnification	Exposure time (s)	Pixel size (μm)	Number of projections over 360°
non-stained kidney	70/4.97	0.39	1	7.6	1601
stained kidney	70/4.80	0.39	1	2.9	1601
stained liver	70/4.38	0.39	2	27.2	2001
stained testicle	50/3.10	0.39	5	7.6	1601
stained testicle	50/3.22	4	10	1.9	1601
stained brain	50/3.01	4	5	4.8	2001
stained heart and lungs	70/3.52	0.39	3	13.6	2001
stained digestive system	70/3.92	0.39	3	20.7	2001

References

[1] Brahm, J. Permeability of Human Red Cells to a Homologous Series of Aliphatic Alcohols. *J. Gen. Physiol.* **81,** 283-304 (1983).

[2] Kumari, S., Roman, A., Khan, A. Chromophore and Spectrum of the Glycogen-Iodine Complex. *J. Polym. Sci. A Polym. Chem.* **34,** 2975-2980 (1996).

[3] Schulz G., et al. Multimodal imaging of human cerebellum - merging X-ray phase microtomography, magnetic resonance microscopy and histology. *Sci. Rep.* **2,** 826 (2012).
